# Resilience and Adaptation: Yukon River Watershed Contaminant Risk Indicators

**DOI:** 10.1155/2018/8421513

**Published:** 2018-10-01

**Authors:** Lawrence Duffy, La'Ona De Wilde, Katie Spellman, Kriya Dunlap, Bonita Dainowski, Susan McCullough, Bret Luick, Mary van Muelken

**Affiliations:** ^1^Resilience and Adaptation Program, University of Alaska Fairbanks, Fairbanks, AK, USA; ^2^International Arctic Research Center, University of Alaska Fairbanks, Fairbanks, AK, USA; ^3^Department of Chemistry and Biochemistry, Institute of Arctic Biology, University of Alaska Fairbanks, Fairbanks, AK, USA; ^4^Interior Alaska Campus, University of Alaska Fairbanks, Fairbanks, AK, USA; ^5^School of Natural Resources and Extension, University of Alaska Fairbanks, Fairbanks, AK, USA; ^6^Resilience and Adaptation Program, University of Alaska Fairbanks, Fairbanks, AK, USA

## Abstract

River watersheds are among the most complex terrestrial features in Alaska, performing valuable ecosystem functions and providing services for human society. Rivers are vital to both estuarine and aquatic biota and play important roles in biogeochemical cycles and physical processes. The functions of watersheds have been used as vulnerability indicators for ecosystem and socioeconomic resilience. Despite a long history of human activity, the Yukon River has not received the holistic and interdisciplinary attention given to the other great American river systems. By using hypothesis-based monitoring of key watershed functions, we can gain insight to regime-shifting stresses such as fire, toxins, and invasive species development. Coupling adaptive risk management practices involving stakeholders with place-based education, especially contaminants and nutrition related, can maintain resilience within communities. The Yukon watershed provides a broadscale opportunity for communities to monitor the environment, manage resources, and contribute to stewardship policy formation. Monitoring keystone species and community activities, such as citizen science, are critical first steps to following changes to resiliency throughout the Yukon watershed. Creating a policy environment that encourages local experimentation and innovation contributes to resilience maintenance during development-imposed stress.

## 1. Introduction

**Figure 1:**
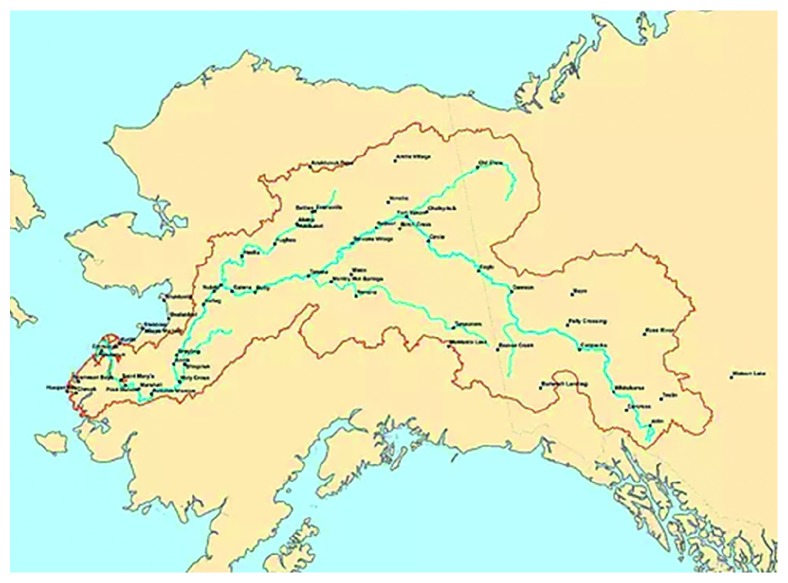
Map of the Yukon River watershed. The Yukon is the third longest river in North America and the fourth largest drainage in North America. https://www.yritwc.org/yukon-river-watershed. Credit: Maryann Fidel, Yukon River Inter-Tribal Watershed Council.

**Figure 2:**
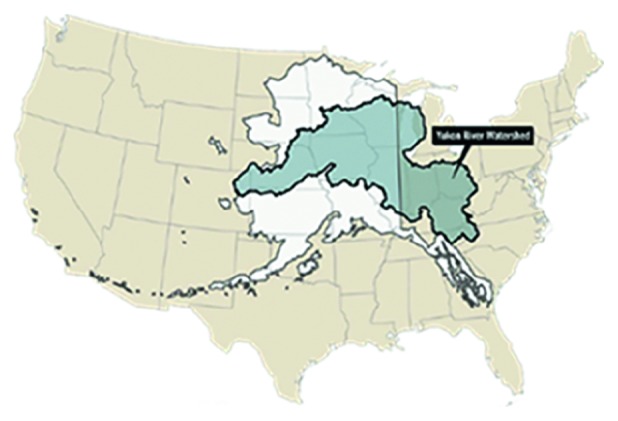
The Yukon River Watershed's footprint compared to Alaska and the United States. Credit: Laris Karklis. Yukon River Inter-Tribal Watershed Council.

There is a long-standing relationship between the Athabascan, Inupiaq, and Yup'ik people living along the Yukon River (Figure 1). The Yukon River provides these communities with salmon, a vital part of their food source. The hunter-gatherers living along this river have been harvesting the five species of Pacific salmon for over 8,000 years. Even today, human activity along the river continues to focus around salmon, such as the salmon fisheries, which also assist in providing the nutritional health, cultural traditions, and ecological integrity. Communities along the river eat several kilograms of subsistence food per week per capita of which 60 percent is fish [1]. The Yukon River drainage is approximately 321,500 square miles and drains about one-third of Alaska (Figure 2). It is roughly 1,980 miles long and is a major transportation corridor for Alaskans living in the state's interior region (Figure 1).

Tribal organizations along the river work to sustain the Yukon River salmon fishing in partnership with state, provincial, and international treaties and laws. The Yukon River Inter-Tribal Watershed Council (YRITWC) is a cooperative network of First Nations and tribes in Alaska and Canada whose goal is to maintain the river as a source of drinking water and healthy salmon and to monitor governmental agencies management of the river. In the past, using art, the tribal groups created images of a healthy watershed. One major theme was evident: a healthy watershed was not just about clean water. Images portrayed beauty, healthy animals, interactive people hunting, and fishing and, in general, a healthy cycle of life. In 2002, the YRITWC initiated the first unified water assessment.

While generally considered clean, the Yukon River watershed has an enduring legacy of pollution resulting from mining and military development. The extraction and processing of metals, coal, and natural gas lead to an increased risk for water degradation due to contaminant release.

Today, pollution also enters the river by global transport form other regions, especially Asia, with its recent activity in energy and economic development [2, 3]. Industrially created persistent organic chemicals and metals are being reported, both in the watershed and its derived food services, such as salmon [1, 4, 5]. The Yukon River is not only important to local and regional ecosystem service issues but also has global economic and industrial applications. The watershed's capacity for resilience serves as an example of an at-risk biocomplex adaptive system tested by both local and global stressors [6, 7].

Due to many components and various interactions of human social systems and ecosystems, there is an ability to change and adapt to new conditions through the generation of new properties [7, 8]. The emergent properties of biocomplexity include self-organization, stability domains, and complex cycles [9]. Viewed on an increasing scale of complexity from individual species to communities, these complex systems may additionally be characterized by stakeholders and regulators (Table 1).

Each level has a characteristic behavior different from the other levels. There are hierarchically distinct behaviors or properties that give the level a property of its own that is greater than the sum of its parts. An effect that is greater than the sum of the individual effects is known as synergism. At higher organizational levels, because the parts are interconnected and their behavior is shaped by feedback loops, adaptive responses to environmental change can occur. As biocomplexity increases, the richness of expression of emergent properties grows, as does the uncertainty. This holistic perspective integrates the human and ecological systems to form a unifying, life-sustaining One Health approach [10]. As a monitoring model, this perspective should tie the selected indicator species to either water or food quality.

Food chains and webs, self-organizing assemblies, are key properties of ecosystems and form a complex system of ecological services. Their ability to change or switch is a property of all complex adaptive systems, such as ecosystems and social systems [7]. Organisms remain in stability domains related to their niche, but can move to a new stability domain; that is, they switch because of an external disturbance into a state of dissolution [11, 12]. Reorganization, leading to transformation, occurs when the system begins to focus around a new stability domain. Chance and outside factors can be important to the course initiated during reorganization. The growth of the human population over the centuries has led to divergent human social systems. In social system cycles, policies can show dramatic change, thus affecting the earth's ecosystems and their management [13]. Overexploitation of a river system, when coupled with climate change, can switch an ecosystem to a different stability domain with loss of some functions [9]. To achieve sustainable and adaptive management in a complex system, we need to be aware of the key aspects of uncertainty: (1) sources of vulnerability, (2) effects of threats of harm, and (3) validity of cause and effect relationships [9]. New policies and frameworks are formulated during reorganization [14]. In this paper, we aim to explore the role and importance of long-term environmental monitoring in supporting the resilience of the Yukon River watershed and its people. We explore frameworks and techniques for aligning stakeholder values in a One Health approach with potential bioindicators of change that could be monitored at different spatial scales.

## 2. Impacts of Scale, Evaluators, and Stakeholders

The environmental impacts of local efforts to provide food, shelter, and clothing for rural communities are much more limited than those of today's economically motivated industries (agricultural, forestry, and textile, for example) that supply large urban centers. The very nature of materials has changed with the advancement of chemical and industrial technologies. However, the vast amount of state and federal lands surrounding the Yukon River still provide space for wildlife and fish and enable Alaska Native communities to practice a traditional hunter-gatherer culture.

Changes in Alaska are becoming more apparent; as populations grow, environmental impacts expand beyond local sites. After recovery, impacts on regional and global systems are often difficult to track and measure. While these are concerns at local and regional levels around the world, the Yukon River has an important position in Alaskan environmental processes. As human activity forces changes to the ecosystem, it is important to consider impacts at different hierarchical scales. Impacts on stakeholders will vary with the stressor involved (Table 1) and, the Yukon River's sustainability, in turn, will depend on its resilience and adaptive capacity [15].

The Alaska Native Claims Settlement Act (ANCSA) is federal legislation enacted to return some control to Alaska Natives and to facilitate disputes between Alaska Natives and the US government. A major outcome of the legislation was the creation of Alaska Native Corporations for economic development [16]. ANCSA brought Alaska Natives into the middle of sustainability conflicts between traditional subsistence hunting and fishing rights, that is, conservation of wilderness, economic development, and a cash economy.

Subsistence is the term most often used to describe a way of life that Alaska Natives have lived for millennia. This way of life, practiced along the Yukon River watershed, is central to individual and community identity, well-being, and sense of place. The government definition of subsistence is more limited and refers to the use of and access to sources of wild food. Conflicts with the cash economy, commercial hunting and fishing, and large mineral development activities have arisen. These environmental practices can have significant impacts on the sources of food and water that have provided ecosystem services maintaining the local community. Often ignored is the loss of aesthetic and cultural value such as sharing and the preservation of resources for future generations [16].

While assessing water security risks faced by river communities is critical, responses must allow for adaptation to management decisions and international policy. Traditional knowledge, with contributions from local residents, integrated with science, provides vital information regarding vulnerability. The relationship between researchers and community members' involvement in research direction empowers all participants by increasing depth of understanding [16].

Mining occupies an historical foothold and remains a growing industry along Alaska's Yukon River watershed. There are still areas, such as the Red Devil Mine (established 1921), that have not been fully remediated. The Donlin Project, also found in the Yukon Kuskokwim region, is one of the largest undeveloped gold resources in the United States. An open pit mine, the Donlin Project, will necessitate the construction of roads and a port on the Kuskokwim River. The development plan also includes the construction of a 315-mile pipeline that will move natural gas from the Cook Inlet. The management of tailings and solid rock waste is of considerable concern. Other major environmental considerations include acid mine drainage, mercury contamination, the use of chemicals for processing, and impacts due to increased road and barge traffic.

## 3. Ecosystem Monitoring and the One Health Approach

Resilience is the capacity to buffer and adapt to new stresses. Management and governance attributes of human systems are tools to navigate the change brought on by the stress. The Yukon River is a socialecological system that is complex and thus will involve uncertainty. The complexity of the Yukon River watershed makes it difficult and expensive to monitor changes in resilience due to its variety of conditions and low population.

One Health is an approach for developing transdisciplinary collaboration across the watershed for the identification and mitigation of health risks in humans, animals, and the environment [10, 17]. The One Health concept derives from an understanding that the health of animals, people, and the environment is connected. One Health is an integrated approach that focuses on the interactions between animals, humans, and their diverse environments. It encourages collaborations, synergies, and cross-fertilization of all professional sectors and actors whose activities may have an impact on health. The One Health concept has special relevance in regions like the Arctic, where people live close to the land and depend upon natural resources for food, medicine, and homemade materials. In Alaska, the One Health approach involves collaborations not only between the human health, veterinary health, and environmental health communities [10] but also with Traditional Ecological Knowledge (TEK) sources in local communities [17].

This special closeness to the land, which is lost in urban communities at lower latitudes, has led to a “One Health” approach to develop an interdisciplinary collaboration that focuses on the interactions between the physical environment, plants, and animals and the human social system to understand the disease processes. The health and survival of rural populations have traditionally depended on an extensive knowledge of the plant and animal species in their local environment. Seasonal harvest and fish migration patterns have traditionally been major community activities that closely link holistic culture and worldview. The local terrestrial, coastal, and marine resources supply natural nutrients and phytochemicals for preventing disease and mitigating metabolic syndromes like diabetes. Understanding the concept of complex processes at both the local and global levels is key to understanding the many varied factors in stress processes for people living in rural, marginal, or polluted environments. One Health emphasizes ecosystem unity. Looking at a map or globe, it is easy to observe populations living in proximity to marine and river ecosystems are at risk from sea level rise, flooding, and toxin disposition [18].

Rivers are vital to both estuarine and aquatic biota and play important roles in biogeochemical cycles and physical processes. Therefore, river watersheds are among the most complex terrestrial features, performing valuable ecosystem functions and providing services for human society. Traditionally, watershed functions are an indicator for ecosystem health [18]. River watersheds have a long history of human activity, but most have not received the holistic and interdisciplinary research attention given other ecosystems. Coupling ecosystem assessment with education creates resilience within the community. By monitoring key watershed indicators such as wildlife, we can gain insight into regime-shifting stresses, that is, increasing contaminants and industrial development (Table 2). Observations of impacts from a changing climate or mineral development can be assessed and inform adaptations to private, state, territorial, tribal, and national ecosystem management approaches [19].

**Figure 3:**
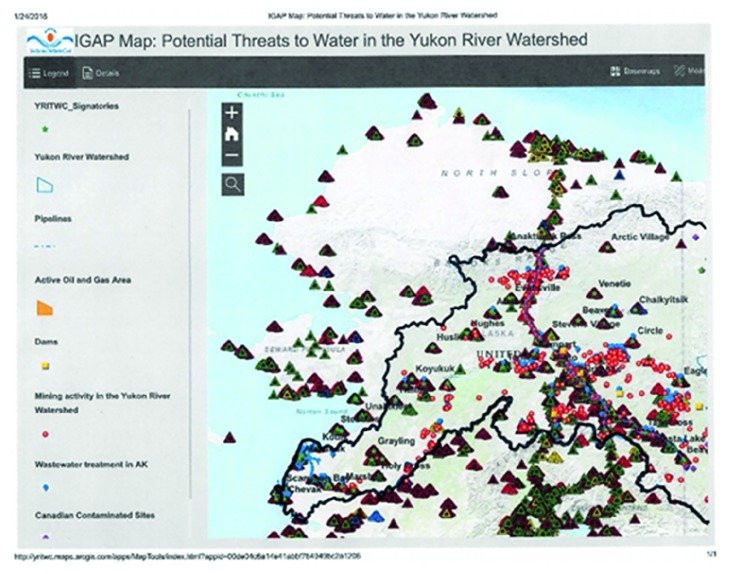
Alaskan potable water sites that are vulnerable and at risk for contamination. Credit: Maryann Fidel, Yukon River Inter-Tribal Watershed Council.

Water and food insecurity are key issues affecting human health (Figure 3). Chemical exposure interferes with metabolic functions and homeostasis by interfering with enzymes or receptors. Increased industrial development at low latitudes and global transport of air pollutants will lead to an increase in contaminant deposition. Legacy chemicals from early development efforts such as gold mining and military construction have been reported in the rural populations. Industrial waste incidents related to development like mining and oil [20] should be monitored until recovery. This process may result in a return to either the previous ecosystem state or the beginning of a reorganization process.

In addition, the tendency of air in temperate regions (some of which is heavily polluted) to warm, rise, and move towards the poles results in Alaska serving as a global sink region for certain kinds of pollution like Hg. The kinds of pollution that have been and continue to be problematic to global health include radionuclides, heavy metals, oil, and many persistent organic pollutants. While the United States does not define carbon dioxide and methane as pollutants, these gasses are the primary waste products resulting from fossil fuel combustion and are contributing to rapid climate change.

In order to understand the local diet, metabolic processes and endocrine function of species occupying a watershed take extended monitoring and research [5]. Even the identification of sentinel species requires knowledge of the ecosystem. There are dozens to hundreds of species in any given food web, and studies have been quite limited along the Yukon River watershed. A true understanding of the health threats of pollution that bioaccumulates and biomagnifies, such as mercury, needs to include data from several trophic levels, beginning with water systems and their security [21, 22]. Sanitation is not well studied in the Yukon watershed (Figure 3), nor is road building [23]; these activities will increase as population and development increase.

The implementation, growth, and development of the Donlin Gold Mine Project, as mentioned earlier, would be a new stress on the relatively pristine Kuskokwim River ecosystem. Using biomarkers at multiple trophic levels to monitor developmental impacts would provide necessary data for revised management policies that could buffer the new stressors. The increased capacity to monitor would allow management to adapt their operation to relieve any unforeseen stress. Extensive monitoring would increase the watershed's resilience and the capacity to buffer and adapt to new stresses.

**Figure 4:**
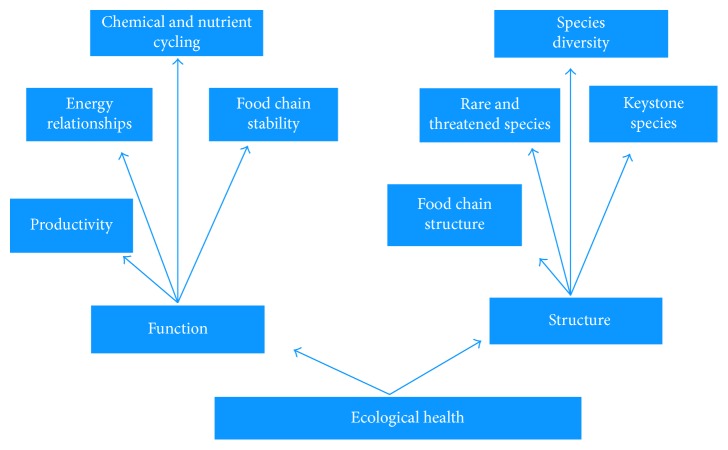
Potential Indicators of ecosystem health.

Another aspect in monitoring ecosystem health, and One Health, is uncertainty. Uncertainty is present at all hierarchical levels and increases as the complexity of the system increases and new system properties emerge. The realization is that material cycling and energy flow are emergent properties of an ecosystem that result from both production and consumption components. Materials move through ecosystems in a cycle of production and consumption. Important elements are carbon, hydrogen, and oxygen, which are required for photosynthesis; and nitrogen, phosphorous, sulfur, calcium, and magnesium, which are required for the construction of proteins and other structural compounds in the bodies of living organisms [22]. These elements are transferred from soil and water to green plants when the plants grow (i.e., production). They are returned to the soil and water whenever carbon chains are broken apart during consumption. Animals and microorganisms are consumers. When consumers derive toxic mineral elements from their food, they retain elements like mercury and pass it up the food chain. Microorganisms are decomposers and consume the bodies of dead plants, animals, and other microorganisms to obtain the carbon-chain building blocks that they need for their growth. As plants grow and die, heavy metals are recycled back into the soil and plants. The growth of all plants in an ecosystem is the ecosystem's net primary production, which is often used as an indicator of function (Figure 4).

The Yukon River flows through regions of permafrost that can accumulate mercury and other potential toxins [24]. While the movement of elements in ecosystems is cyclic in a closed system, the movement of energy is not cyclic. Energy enters ecosystems as sunlight, and the energy is used by photosynthesis to create carbon compounds. When consumers use the carbon chains in their food as building blocks for their bodies, they breakdown some of the carbon chains to release energy for their metabolic needs. After consumers use the energy from respiration and the producers and consumers die, there is a flow of metals and persistent organic toxins through aquatic ecosystems [25–28].

The indigenous peoples of the circumpolar North are secondary consumers, quite literally at the top of the trophic food web [17, 29]. While the physical benefits of subsistence living may still outweigh the risks of contaminants, health risks may become health problems unless there are improvements in the monitoring of chemical waste. In the last decade, the international community agreed to work towards the elimination of bioaccumulative toxic contaminants. The United Nations has crafted several international treaties for environmental protection. The Stockholm Convention is among those treaties that use precautionary language [4]. The Stockholm Convention was designed to eliminate or restrict the production and use of persistent organic compounds. While chemical pollution will inevitably be an issue for decades to come, implementation of the Stockholm Convention should be a major step toward identifying the toxic contaminants that need monitoring in the Yukon River watershed. In addition to climate change and industrial pollution, the Yukon River watershed residents are experiencing a nutritional transition in dietary and lifestyle patterns [30]. The combination of increased contaminants and changing dietary patterns suggests that more frequent monitoring of the watershed's social ecological system would provide a finer scale of information for policy-makers [7].

## 4. Bioindicators for Monitoring Arctic and Subarctic Air, Water, and Food Systems

Subtle changes in the environment that affect vital resources such as air quality, the quantity or quality of fresh water, and the distribution and assemblages of native and introduced species will have profound effects on human, animal, and plant health. There have been many examples of the disastrous, unintended effects of human actions. The study of the feedbacks between human health and the environment must therefore be comprehensive, including research on the long-term regime shifts associated with climate change [31, 32].

Contaminants associated with air and water create a serious health problem in the Arctic because they strongly affect both the city life and traditional way of life of the indigenous peoples [26, 27]. The contaminants have a tendency to accumulate in certain animals, especially in marine mammals, caribou, and moose used as traditional food by indigenous people. The exposure to contaminants is closely connected to local consumption of traditional food. Burger and colleagues [16] have suggested that bioindicators can be used to monitor the changes in the status of ecosystems or to evaluate remediation efforts. Bioindicators usually refer to organisms and their properties or functions as components of an ecosystem (Figure 4). Both the ecosystem and its stakeholders benefit by relevant monitoring at different scales (Table 2) by allowing for adaptive management of contaminants and engagement in policy creation.

Healthy ecosystems need to be maintained to ensure they receive services form the ecosystems. Bioindicators that indicate both organismal and human health are desirable [33]. Carnivores, such as river otters, mink, fox, and other predators, have been useful bioindicators. There is a need to select common and widespread species as bioindicators because their populations can be monitored and stresses compared between regions. Bioindicator species provide insight into both the functional and structural aspects of a healthy ecosystem. Some common indicator species at the different hierarchical levels are listed in Table 2.

Predatory species that live at a higher trophic level are more comparable to humans and thereby more exposed to biomagnification, such as mercury [1, 34, 35]. For example, river otters, fox, dogs, and humans all eat fish. Indicator species lower on the food chain can be used to monitor potential damage to higher trophic level organisms within ecosystems.

## 5. Examples

### 5.1. Lichens: As Monitors of Global Transport

Lichens connect air, water, and environment. Fungi and green algae form symbiotics called lichens. Lichens are a symbiotic association of algae (phycobiont) and fungi (mycobiont), and are long lived, growing on rock, wood, or soil substrates. They extract water and labile nutrients from the air and are therefore sensitive to air pollution. Among the many mammal and bird species having potential uses for biomonitoring, *Rangifer* spp. (caribou and reindeer) are particularly important because they continue to serve as a food staple across the circumpolar north. Reindeer and caribou are associated with boreal forest, taiga, and tundra biomes in which the major components of their summer diet are low-growing species such as sedges, willows, and lichens, while lichens are the main component in the winter diet [36]. These diet regimes can be impacted by climate change factors, such as temperature and precipitation by reducing the growth of lichens, which are replaced by sedges through competitive interactions. Yukon watershed environs may be more impacted than temperate regions by melting permafrost and flooding [24, 25, 28].

### 5.2. Salmon: As a Key Component of Ecosystem Services

Salmon are a key component of the Yukon River watershed and provide insight into both the functional and structural aspects of this river system. Monitoring should be coordinated with the communities and the YRITWC. Salmon provide nourishment services to the people, animals, and the land. Alaskans depend on salmon for subsistence and employment; spawners feed bears and birds and enrich the soil. The Yukon River has migrations of all five species of Pacific salmon.

Salmon run strength is monitored by both community residents and state and federal agency personnel. Monitoring salmon runs during declines is essential to understand the dynamics of the system. Population declines have sometimes led to restoration polices that aim to restore run strength. However, restoration practices may introduce excess nutrients, disease, and toxic substances. Restoration activities involving nutrients must be balanced and monitored to prevent harm caused by increased toxic loads [26].

Human health benefits are related to the intake of omega-3 fatty acid which is obtained through consumption of oily fish, such as salmon [37]. The positive health benefits of salmon can be reduced by accumulation of environmental pollutants such as mercury, lead, or persistent organochlorine contaminants. Dietary exposure to contaminants can increase the risk of cancer, immune dysfunction, diabetes, and effects on the aging process [38].

The people in the small, rural communities along the Yukon River and its tributaries rely on salmon for nourishment. The cost of country store foods should be surveyed to monitor changes in price on a routine basis.

### 5.3. Red Foxes Can Be a Pan Arctic and Sub Arctic Food Chain Sentinel

Red foxes (*Vulpes vulpes*) are a widespread species and opportunistic omnivores who inhabit most ecosystems across the Arctic and subarctic. Their feeding ecology can inform the interpretation of contaminant uptake patterns when the trophic level is characterized. Stable isotope studies traditionally trace pathways of organic matter using stable carbon isotopes which are reflective of naturally occurring isotope values in the source of the terrestrial animals' diet [39]. Besides terrestrial sources, stable isotope ratios can also reflect dietary sources from coastal versus noncoastal, bethnic versus pelagic environments (i.e., food webs). Studying the diet composition by using stable isotope analysis allows a view of the diet over different time frames, because of the different turnover rates for different tissues and continuous growth of certain structures. For example, when analyzing the pelage cycle of hair, mammals like the red fox (*Vulpes vulpes*) will lose hair in late spring, approximately in May, and regrowth occurs through October. Fox hair will be reflective of a diet consumed during late summer through October [40]. The stable isotope ratio of nitrogen has been used to establish trophic levels [39] and food web structures. In an ecosystem, most organisms consume more than one kind of prey; therefore, a network of overlapping food webs will influence what trophic level an animal is occupying. Generally, at each trophic level, the nitrogen isotope ratios of the predator increases in natural food chains [41]. There are, however, limitations with interpreting these trophic level interactions when they are determined only from stable isotope data; for example, the age and health status of the animal [42, 43]. The average life span of a red fox is five years in the wild; the animal is sexually mature at around 10 months [5]. The red fox may move north into the Arctic fox range following development of road systems [44].

### 5.4. Sled Dogs as a Human Health Sentinel

Dogs have become a popular model for immune function, nutrition, exercise, toxicology, and cognitive disorders [45–49] because they have key features associated with cognitive dysfunctions, beta-amyloid pathology, and oxidative damage similar to that of humans [47]. Sled dog mushing, once used primarily as a means of transportation around the Yukon River watershed, has evolved into a popular national and international sport. Sled dogs are unique research models because the effects of diet, exercise, disease, and environment can be observed on the immune, cardiovascular, and endocrine systems and their indicator biomarkers.

Sled dogs in northern climates are often exposed to the same environmental hazards as their human counterparts [35]. In many Alaskan villages, sled dogs are still a fundamental part of a traditional lifestyle, used for trapping, packing, and transportation. Most of these villages are small settlements, established on or near rivers to facilitate travel and food gathering. The diet of both Alaska Natives and their sled dogs often comprises a variety of berries, wild game, fish, and marine mammals [34]. Before the arrival of western diets, circumpolar people had a low incidence of obesity, diabetes, and cardiovascular disease. Researchers attribute this to the loss of a typical subsistence diet, abundant in polyunsaturated fatty acids and antioxidants [50]. Sled dogs provide a large homogeneous sample size for studying the impact of subsistence lifestyles and subarctic environments on immune and endocrine functions. Since village canine populations live in close association, they are considered good sentinels for environmental pollutants [48, 49].

## 6. Adaptation and Resilience

Alaska is rich in biological, physical, and intangible resources. Millions of animals (birds, fish, and marine mammals) migrate to the Arctic to reproduce. These resident and migrating animals feed thousands of people dispersed in small communities throughout the circumpolar North. Several nations also have significant oil and mineral deposits in the Arctic. In addition, the Far North also has tremendous strategic importance and the space needed for military training and testing. Therefore, the migratory nature of many species and their shared ecosystems (the Arctic Ocean and the boreal forest) in the Earth's largest remaining regions of the Arctic wilderness will require resource management in the Arctic to operate at an international scale. All over the world, the idea of stewardship is being reinvestigated with new understandings of complexity and recognition of past adverse effects on watersheds. There has been some improvement, as over the last two decades, adaptive management has grown as a model for stewardship and resilience [7].

**Figure 5:**
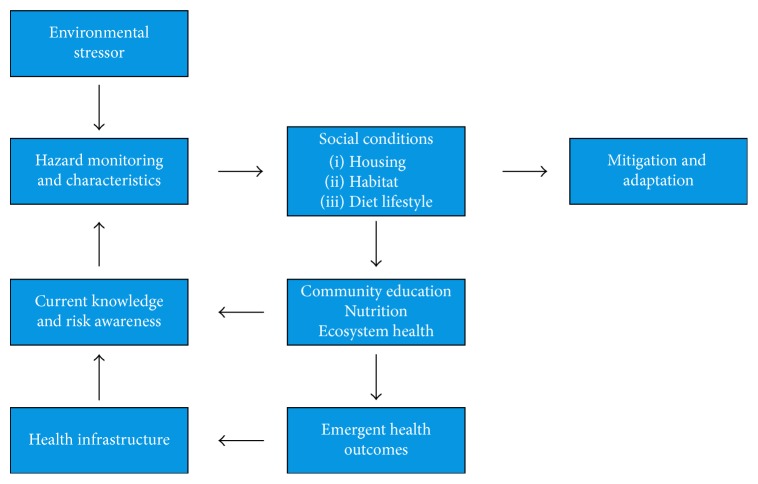
Resilience framework related to monitoring environmental stressors.

Running through the boreal forest, the Yukon River watershed has a long history of human activity but has not been given the holistic and interdisciplinary research attention in the same manner as the other great North American river systems. Using hypothesis-based monitoring of key watershed functions, we can gain insights to regime shifting stresses such as increasing river carbon transport, food system diversity, contaminant load, and the effect of fire on subsistence plants, animals, and human social structure. These stresses could impact the river's biogeochemical organization, especially the movement of toxins, contaminants, and waste. Bioindicators will require an important effort to monitor change resilience in Alaska's watersheds and estimate their degree of resilience in the future [7]. Additionally, by including students and citizens in the monitoring plan, it becomes more holistic by measuring impacts on the various “capitals” that may be impacted (Figure 5). Red foxes, river otters, mink, and even dogs have been used as bioindicator species to monitor variation in mercury concentrations both within a species and between different species living in a local geophysical region or ecosystem [5, 21, 34, 51]. Foxes can provide information on Hg biomagnification patterns and changes in exposure. Since foxes are omnivores, like dogs, examining concentrations of Hg also provide feeding ecology data on contaminant concentrations in other small mammals, birds and fish.

A major goal should be to develop a holistic framework that defines resilience. Resilience can maintain stability or adapt and transform to new regimes [52, 53]. Regime shifts can increase the risk of further regime shifts and lead to a cascade of regime shifts, a tipping point, to a very different state [8]. To monitor components of resilience in the Yukon River watershed, the development of appropriate local ecological and social indicators is needed to assess aspects of adaptive capacity and management (Tables 1 and 2). The ability of the river system to adjust to changing conditions, such as physical impacts of climate change or social desire for development, will depend on the degree of potential self-organization, flexibility of action, and avoidance of institutional “one size fits all” solutions. All sources of knowledge must be respected to maintain or increase resilience. People living in the region, some for many generations, have knowledge that can lead to sustainable solutions. Traditional ecological knowledge is a means to increase both resilience and adaptive capacity as the Yukon watershed is impacted by the Anthropocene [54, 55].

**Figure 6:**
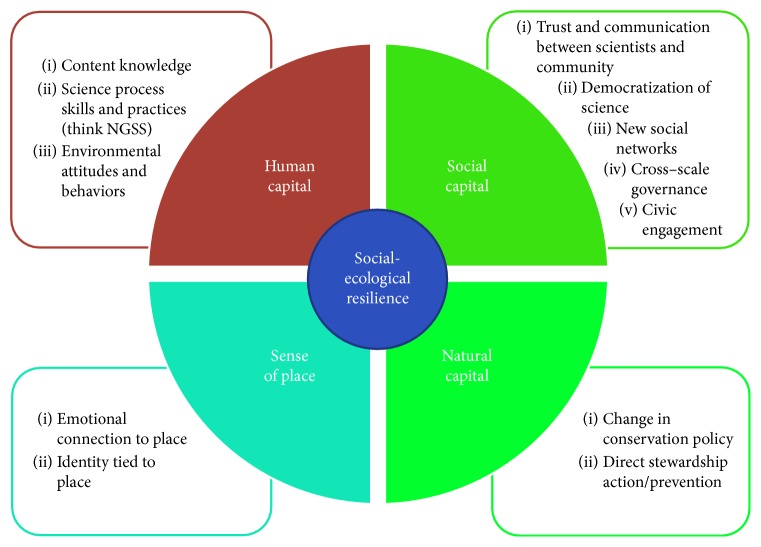
Citizen science and community-based environmental monitoring contribute to socialecological resilience by building human capital, social capital, natural capital, and sense of place. Documented outcomes in each category are summarized from the review conceptualized by Spellman [58].

Monitoring of the Yukon River system for contaminant changes can lead to activation of a mitigation program. Such a mitigation program would consist of contaminant removal, development of a robust social support system, and, if necessary, nutritional replacement [8]. Mitigation programs that include education programs and stakeholder participation reduce the impact of uncertainty and enhance the resilience of the entire watershed (Figure 5). Citizen science or community-based monitoring programs are methods that include stakeholders and emphasize education alongside environmental monitoring [58]. These types of programs have a wide range of documented outcomes that can contribute to the watershed's resilience (Figure 6). Yukon River watershed users may eventually face the decision of whether or not to consume wild fish. Toxic metals and persistent or organic pollutants are risks that need to be considered in relationship to the benefits provided by omega-3 fatty acids, vitamins, and other beneficial nutrients found in fish. Detailed, local-scaled monitoring, identification of thresholds, and community education will be needed [55]. Future nutritional transitions have implications for individual and community health.

Mercury in the Arctic is an example of a significant environmental and human health issue. Northern people's reliance on local, traditional foods increases their risk of mercury exposure [24, 56]. Increased mercury mobility is a major impact of climate change. Sea-level changes and flooding events in high latitude coastal ecosystems, for example, will increase the bioavailability of contaminants such as mercury [57]. Mercury concentrations have been used as an indicator of past exposure to heavy metals in ancient fish and animal samples. Sea otters (*Enhydra lutris*) and river otters are common to the Gulf of Alaska's coastal areas and paleontological deposits of their bones have been identified as far back as the early Holocene. Stable isotope ratios are used to reconstruct ancient food webs and help identify sea otter prey, which may have bioaccumulated high concentrations of the metal. Modern sea otters have *δ*^13^C, *δ*^15^N, and mercury values corresponding largely to a benthic diet. Higher *δ*^15^N and mercury levels were found in ancient sea otter bones which may demonstrate higher trophic level foraging and a large increase in the bioavailability of mercury in the coastal ecosystem. These large increases may be associated with rising sea level after the glacial maximum. Sea otter paleontological remains can be used to place present day predicted climactic perturbations, like flooding of Beringia during the Holocene.

At this time, proper monitoring and analysis of the Yukon River watershed is missing. Individual researchers from federal and state agencies as well as the University of Alaska are studying individual issues related to governmental objectives. Resilience is sometimes discussed, but its concepts are relatively new and adaptive management is not universally used. Linking the concepts of resilience and the people power in citizen science is a potential way to accomplish a holistic study. Currently, during this early period of stress from development and climate change, the appropriate studies are not occurring. This is an opportunity to test the generalities and ecological concepts on a watershed that has not yet been reduced by humanity.

In adapting to the impacts of development and climate change, state and federal managers need to monitor keystone species so that watershed resilience can be tracked. When choosing a keystone species, place-based factors should be considered equally with the more broad geographic distribution of the plant or animal. In a region as large as the Yukon River watershed, it is imperative to recruit citizen scientists to monitor the selected ecosystem and social health indicators to inform local policy and provide data for adaptive management [58, 59].

## 7. Conclusion

River watersheds are among the most complex terrestrial features in Alaska performing valuable ecosystem functions and providing services for human society. Rivers are vital to both estuarine and aquatic biota and play important roles in biogeochemical cycles and physical processes. The Yukon River watershed has a long history of human activity but has not been given the holistic and interdisciplinary research attention of the other great American river systems. There is a need to identify relationships and potential feedbacks between the Yukon River watershed's health and human health. There is a need to increase place-based education in rural communities to increase the resilience of the river system. The Yukon River watershed provides a broadscale opportunity for resilience baseline studies to determine the impact of stress on ecosystem services. Monitoring adaptive capacity and increased resilience will enable managers and community members to modify watershed policies.

## Figures and Tables

**Table 1:** Types of river resources and their value to potentially impacted stakeholders.

Type	Definition	Examples	Stakeholders
Individual resources	Value of individual species by its trophic position	Keystone species	Natural resource trustees
		Trophic role	Conservationist
		Endangered species	Regulators
			Tribal councils
			Scientists

Specific resources for individuals	Value of individual species to individual people or groups of people	Fish, game, and sport activities	Fish and game agencies
		Wildlife for photography; birdwatching	Conservationists
		Plants for religious or medicinal purposes	Businesses catering to recreationists
			Tribal councils
			Scientists and social scientists

Resources for communities	Value of ecosystem to human communities	Clean water and air	Regulators
		Habitat	Public policy-makers
		Subsistence species	Coastal zone managers/Army Corps of Engineers
			Tribal councils

Intact ecosystems	Ecological, aesthetic, and existence values to people	Tundra	Environmental protection agencies (state and federal)
		Boreal forests and rivers	NGOs and tribal councils
		Rivers	

**Table 2:** Potential indicators of change and variation across ecological levels.

Ecological level	Indicators/metrics	Rationale
Individual species	Salmon	Common; abundant; widespread; eaten by higher trophic levels
	Shellfish	
	River otter, sea otter, mink, and muskrat	Represents higher trophic levels; monitors different food webs

Populations	Colonial birds	Of interest to the public, vulnerable because they breed and feed in aquatic habitats that concentrate toxins
	Red fox and Arctic fox	Of less interest to the public, but have circumpolar perspective

Community	Sled dogs	Of interest to the public, vulnerable because they eat what humans eat
	Lichens and plants	Low trophic level and thus indicative of higher level effects; monitors global transport

Ecosystem	Species diversity	Of interest to the public, can be used to observe trophic dynamics

Landscape	Percent habitat	Of interest to the public, used to assess quality of habitat as well as temporal changes
